# Challenges in the detection and assembly of virus integration structures in human genomes

**DOI:** 10.3389/fonc.2026.1925720

**Published:** 2026-09-14

**Authors:** Xinyi Deng, Xiaomeng Du, Elizabeth Gensterblum-Miller, Jordan Currie, Behirda Karaj Majchrowski, Penelope Lialios, Apurva Bhangale, Matthew E. Spector, Alan P. Boyle, J. Chad Brenner, Ryan E. Mills

**Affiliations:** 1 Department of Computational Medicine and Bioinformatics, University of Michigan Medical School, Ann Arbor, MI, United States; 2 Department of Otolaryngology-Head and Neck Surgery, Michigan Medicine, Ann, Arbor, MI, United States; 3 Cellular and Molecular Biology Program, University of Michigan Medical School, Ann Arbor, MI, United States; 4 Department of Pharmacology, University of Michigan, Ann Arbor, MI, United States; 5 University of Pittsburgh Medical Center, Pittsburgh, PA, United States; 6 Rogel Cancer Center, University of Michigan, Ann Arbor, MI, United States; 7 Department of Human Genetics, University of Michigan, Ann Arbor, MI, United States

**Keywords:** genome assembly, HPV, Oxford Nanopore sequencing, structural variation, viral host integration

## Abstract

Oncogenic viral infections are major contributors to cancer development worldwide. Tumor-associated viruses such as human papillomavirus (HPV), hepatitis B virus (HBV), Epstein–Barr virus (EBV), and Merkel cell polyomavirus (MCPyV) can promote malignant transformation through diverse mechanisms, including persistent viral gene expression, chronic inflammation, and, in some cases, integration of viral DNA into the host genome. Among these, HPV is one of the most clinically important DNA tumor viruses and is a major driver of cancers of the cervix, anus, penis, vagina, vulva, and oropharynx, collectively accounting for over 400,000 deaths annually (1). In infected cells, HPV can persist as episomal DNA or integrate into the host genome. Importantly, HPV integration plays an important role in tumorigenesis and often generates complex viral–host genomic rearrangements that are difficult to resolve using conventional short-read sequencing approaches. Long-read sequencing technologies offer new opportunities to reconstruct these intricate integration structures, but the performance of existing assembly strategies remains incompletely evaluated. In this study, we systematically review sequencing platforms and their application for detecting structural variants and evaluate long-read assembly tools for reconstructing HPV integration structures. Using three synthetic Oxford Nanopore DNA sequencing datasets representing different levels of integration complexity together with the UMSCC47 cell line as an authentic long-read sequencing dataset, we assess whether structural-variant detection methods and genome assembly can accurately identify complex integration structures, particularly under conditions of high copy number and structural rearrangement. Our results provide practical guidance for selecting sequencing technologies and computational approaches for viral integration detection and structural resolution, enabling a more comprehensive understanding of virus-driven genome remodeling in cancer.

## 1 Introduction

Viruses are causative agents in several human cancers, with known tumor viruses including Epstein–Barr virus (EBV), hepatitis B virus (HBV), hepatitis C virus (HCV), human papillomaviruses (HPVs), human T-lymphotropic virus 1 (HTLV-1), Kaposi sarcoma-associated herpesvirus (KSHV; also known as human herpesvirus 8 (HHV-8)) and Merkel cell polyomavirus (MCPyV or MCPV) (**Figure 1A**). While chronic infection and constitutive viral gene expression from some oncogenic viruses are sufficient to induce tumorigenesis in several of these diseases, viral genomic integration into the host DNA is a contributing and/or causative factor. For example, in Merkel cell carcinoma caused by MCPyV, integration sites are clonally identical, suggesting integration occurs prior to tumor expansion (1). Another example is HPV-associated cancer. High-risk HPV types, which collectively account for over 400,000 deaths annually (2), are present in 70% of oropharyngeal carcinomas (3) and >99% of cervical carcinomas (4), are frequently integrated as well, and integration is thought to be a major factor in the progression and invasiveness of cervical cancer (5).

**Figure 1:**
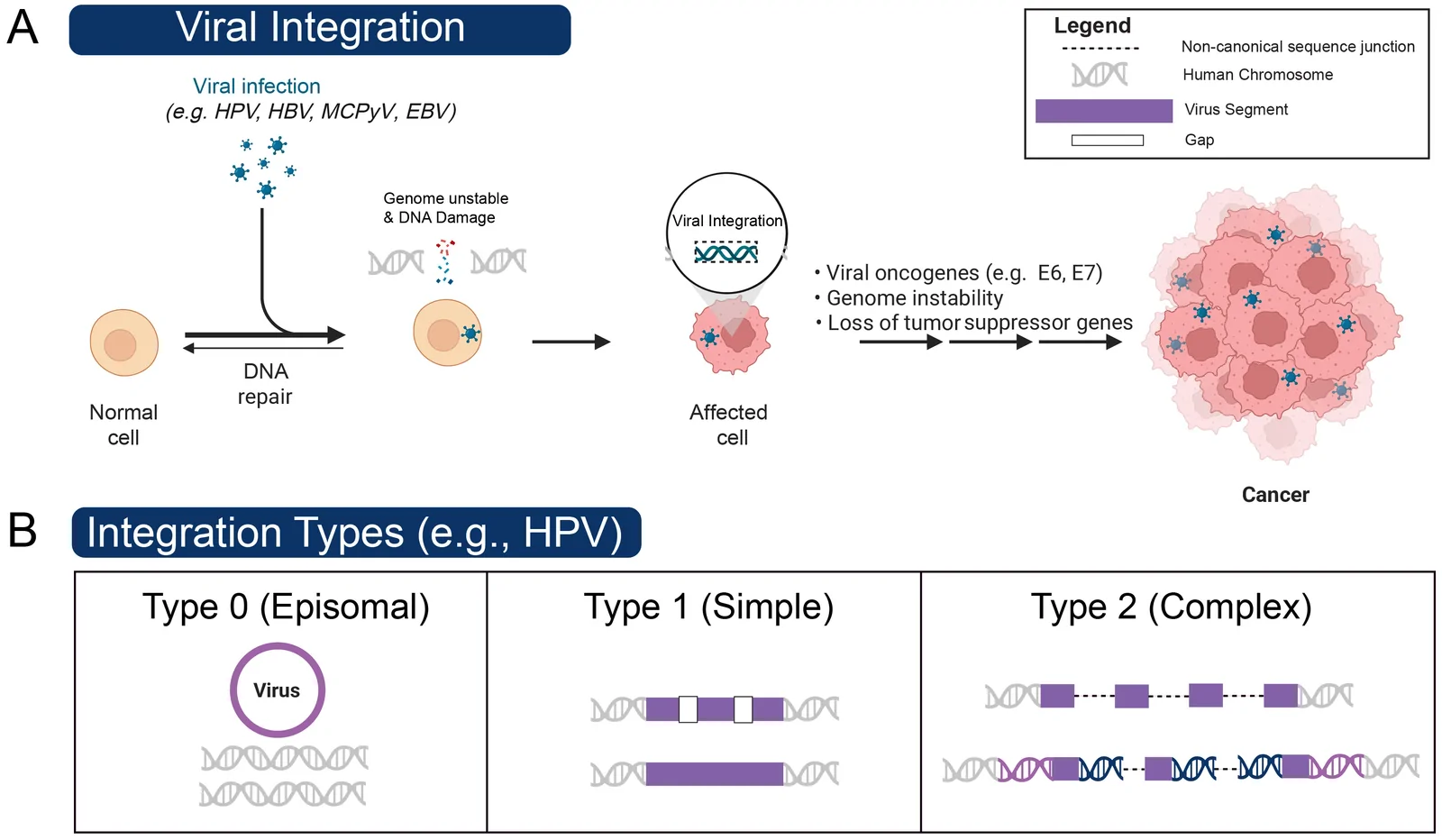
Viral integration process during tumor development and integration type classification. **(A)** Viral infection can contribute to tumor development through changing viral oncogene and tumor suppressor gene expression, causing genome instability, and integration of viral DNA into the host genome. **(B)** Viruses may persist as episomal DNA or become integrated into host chromosomes during cancer progression. Viral-positive tumors, for example HPV-positive tumors, are commonly classified into three integration patterns: Type 0, with no detectable viral integration and viral genomes maintained in an episomal state; Type 1, simple integration involving a limited number of viral-host junctions, where the integrated viral fragment may include partial viral sequences or contain deletions; and Type 2, complex integration characterized by multiple viral-host breakpoints, copy-number alterations, or structural rearrangements. This schematic summarizes the transition from viral infection to integration-associated genomic remodeling and highlights the major categories of HPV integration structures. Some schematic elements were created with BioRender.com.

Based on viral genome status and integration complexity, virus-positive tumors can exhibit episomal, simple integration, or complex integration patterns. Viral-positive tumors lacking detectable integration are commonly referred to as episomal (6). Here, we designate episomal viral genomes lacking detectable host integration as Type 0 in **Figure 1B**. Taking HPV as a well-characterized example, integrated viral genomes are generally classified as Type 1 (simple integration) or Type 2 (complex integration) (7–9) (**Figure 1B** Type 1 and Type 2). Viral integration can influence cancer development and progression through a variety of mechanisms. In its simplest form, integration ensures continued replication of the viral genome as the tumor expands, encoding viral gene products (both protein and Non-coding RNA (10)), which may serve to disrupt critical tumor suppressor functions. Indeed, HBV, HCV, HPV, and MCPyV all encode proteins that interact with and disrupt the function of p53 (11, 12). The integration process itself may also disrupt viral regulatory genes. In high-risk HPVs, the HPV16 genome encodes early (E) and late (L) genes with distinct functions in the viral life cycle and oncogenesis. Early genes, including E1 and E2, regulate viral replication and transcription, while E6 and E7 act as oncogenes that disrupt key pathways. Late genes (L1 and L2) encode structural proteins required for viral capsid formation. Integration commonly disrupts E1/E2 expression, which functions to regulate and suppress E6/E7 expression, leading to their unchecked expression (13). Viral sequences may also integrate within genic regions, leading to partial or total loss of gene function. This has been documented with HPV16 insertion into the tumor suppressor gene castor zinc finger 1 (14) as well as some recurrent targeting of several cancer genes, such as *PDL1* and *MYC* upon HPV integration, that are related to oncogenes and immune checkpoints (15).

Viral integration is thought to most commonly occur via repair pathways following DNA double-stranded breaks, with a preference for non-homologous end joining (NHEJ) or microhomology-mediated end joining (MMEJ) varying among different viruses. Integrations usually combine with multiple copies of all or parts of the genome integrated, resulting in complex structural rearrangements (16) (**Figure 1B**). Local clusters of multiple host-viral breakpoints are associated with somatic copy number alterations in HPV+ Cervical squamous cell carcinoma and endocervical adenocarcinoma (CESC) and head and neck squamous cell carcinoma (HNSCC) tumors. These regions are enriched for enhancers, and their amplification can form super-enhancer structures that increase the expression of the viral oncogenes (17). In addition to the co-amplification of host regulatory sequences, viral integration is also associated with the duplication of proximal genes. Multiple tumor viruses have been associated with duplication and subsequent overexpression of MYC [HPV16 and HPV18 - cervical cancer (16, 18, 19); HBV - hepatocellular carcinoma (20)]. While these examples demonstrate documented cases of insertional mutagenesis near a driver oncogene, in general integrations are preferentially associated with genomic fragile sites and coding regions broadly, not specific oncogenic hotspots (21, 22). Thus, the ability to determine the integration landscape of a given tumor without prior sequence knowledge holds promise for predicting disease severity.

The diverse nature of viral integration patterns and their downstream molecular effects necessitate the ability to map integrations at the individual integration structure level. Challenges to this goal include the long length of some tumor viruses (e.g., EBV > 172 kbp) (23), the complexity of viral integrations, and the presence of integrated, episomal, and extrachromosomal (24) copies within a single tumor, all of which complicate analysis. While short-read whole genome sequencing (srWGS) has facilitated critical discoveries in the field, such as defining MCPyV as the causative agent of MCC (1), further progress will require the incorporation of long-read sequencing technologies and novel genome assembly tools that are designed with these challenges in mind.

In this review paper, we provide an overview of the available sequencing technologies and computational frameworks for reconstructing complex viral integration structures. We also discuss the current limitations of these approaches and highlight future methodological and technological developments needed to improve reconstruction accuracy.

To systematically address these challenges, we first review short-read sequencing approaches and their limitations, followed by an overview of long-read sequencing technologies and structural-variant detection methods. We then focus on assembly-based strategies for resolving complex HPV integration structures, with particular emphasis on ONT long-read data and collapsed assembly frameworks. We selected HPV integration as a representative benchmark because HPV has a compact genome (~8 kbp) but frequently forms complex integration structures containing tandem viral repeats, concatemers, and rearranged virus–host junctions. These repetitive structures are particularly prone to assembly collapse and misassembly. In contrast, EBV contains substantially larger unique genomic regions that facilitate assembly (25), whereas HBV and MCPyV integrations are typically less structurally complex (26, 27). Consequently, HPV integration provides a stringent test case for evaluating the ability of assembly algorithms to reconstruct complex virus–host genomic structures, and our results would thus be likely applicable to other genomic viral integration scenarios.

Based on this design, we ask whether traditional whole genome assembly tools and structural-variant detection methods can correctly identify complex HPV integration structures, especially when the copy number is high or when rearrangements are present. To evaluate this, we include three synthetic Oxford Nanopore DNA sequences with different levels of HPV integration complexity, as well as a long-read dataset from the well-characterized UMSCC47 cell line.

## 2 Sequencing technologies for viral integration analysis

Genome sequencing technologies have evolved substantially over the past few decades, progressing through multiple generations of innovation (**Table 1**). First-generation sequencing, represented by Sanger sequencing, relies on chain termination using dideoxynucleotides (ddNTPs) to generate DNA fragments of varying lengths, from which the sequence is inferred. The development of short-read whole genome sequencing (srWGS), also called next-generation sequencing, introduced massive parallelization of sequencing reactions, enabling high-throughput sequencing of millions of fragments simultaneously. This dramatic increase in throughput, coupled with a reduction in cost per base, has facilitated not only whole genome sequencing but also large-scale profiling of the transcriptome, epigenome, and exome through various enrichment and conversion strategies. More recently, long-read whole genome sequencing (lrWGS), also called third-generation sequencing have enabled long-read, single-molecule, real-time sequencing. The most widely adopted platforms include those developed by Oxford Nanopore Technologies (ONT) and Pacific Biosciences (PacBio), which provide substantially longer read lengths and improved capabilities for resolving complex genomic structures.

**Table 1:** Sequencing platforms for viral integration detection.

Category	Sequencing platform	Typical read length and accuracy	Enrichment strategy	Major strengths	Major limitations	Most suitable application in viral integration analysis
Short-read Whole Genome Sequencing	Illumina (28), Ion Torrent (29), MGI(BGI) (30), Ultima Genomics (31)	~50–400 bp; high per-base accuracy	genome-wide sequencing	High throughput, relatively low cost, mature pipelines; detects integration using discordant read pairs, split reads, and chimeric reads	Limited resolution of repetitive regions, large SVs, viral concatemers, and complex rearrangements	Large-cohort screening and initial discovery of viral integration sites
Long-read Whole Genome Sequencing	PacBio HiFi WGS (108)	~10–25 kbp; high consensus accuracy, often >99.9%	genome-wide sequencing	High accuracy long reads; strong for haplotype-resolved assembly, breakpoint localization, and complex structural reconstruction	Higher cost and lower throughput than short-read WGS; requires high-quality DNA	Accurate reconstruction of viral-host integration architecture and complex SVs
	Oxford Nanopore Technology (ONT) WGS (109)	~10 kbp to >1 mbp; ~95-99% depending on chemistry/basecalling	genome-wide sequencing	Ultra-long reads can span large integration loci, tandem duplications, viral concatemers, and repetitive regions	Higher raw error rate than PacBio HiFi; results depend on basecalling and alignment quality	Resolving large-scale or complex viral-host genomic structures

	Types	Sequencing platform	Enrichment strategy	Major strengths	Major limitations	Most suitable application in viral integration analysis
Target capture strategies (used for short and long reads)	Probe-based capture	Illumina capture panels, Twist Bioscience panels, nanopore Cas9-targeted sequencing (nCATS) (42), and Twist ONT (43)	pre-designed synthetic probes	Increases sensitivity for low-abundance viral sequences; improves coverage at selected regions	Limited to probe-designed regions; may miss unexpected or highly rearranged sequences	Sensitive detection of known or expected viral integration sites
	real-time capture	Nanopore-based approaches: Readfish (44), UNCALLED (45)	real-time analysis of partially sequenced reads and selectively accept or reject DNA molecules based on alignment to a reference genome	Flexible enrichment without probe synthesis; target set can be updated computationally	Enrichment depends on real-time mapping and reference quality	Rapid enrichment of viral genomes or candidate integration regions

To further improve sensitivity for low-abundance targets, enrichment-based strategies such as target capture sequencing have been widely adopted. In these approaches, hybridization probes are used to selectively isolate genomic regions of interest before sequencing, thereby increasing coverage of specific loci, such as viral genomes or integration sites. Alongside these technological advances, our ability to detect and resolve complex viral integration and structural variation has improved substantially, enabling more accurate reconstruction of viral–host genomic architectures.

### 2.1 Short-read whole genome sequencing

SrWGS technologies have played a central role in the discovery and characterization of tumor virus integration. Widely adopted platforms such as Illumina (28) and other short-read sequencing systems such as Ion Torrent (29), MGI (BGI) (30), and Ultima Genomics (31) have enabled large-scale genomic studies. Illumina sequencing, based on sequencing-by-synthesis (SBS) chemistry, is the most widely adopted short-read sequencing platform. This approach uses four fluorescently labeled reversible terminator nucleotides, allowing the incorporation of a single base per cycle, which is detected through imaging. The sequencing workflow involves library preparation with adapter ligation, followed by bridge amplification on a flow cell to generate clonal clusters, enabling massively parallel sequencing of millions to billions of DNA fragments simultaneously. This technology provides high accuracy and throughput, typically generating short reads ranging from 50 to 300 base pairs.

These short reads provide the foundation for detecting structural variation through characteristic alignment patterns, including discordant read pairs, split reads, and chimeric alignments. Such signals enable the identification of genomic rearrangements as well as virus–host junctions, forming the basis for viral integration site detection. Many computational tools have been developed for this purpose, including SurVirus (32) and searcHPV (33).

However, all these technologies share a fundamental limitation: their inability to sequence long contiguous DNA fragments. Consequently, the detection of structural variants (SVs) and the assembly of repetitive or duplicated regions are often hindered by short-read lengths. These regions include segmental duplications, long interspersed nuclear elements (LINEs), short tandem repeats (STRs), variable number tandem repeats (VNTRs), telomeres, and satellite DNA, which can extend to tens of megabases. Importantly, such repetitive elements are commonly associated with HPV integration loci, posing additional challenges for accurate breakpoint detection and structural reconstruction. From the SV detection aspect, these regions are difficult to characterize because short reads often cannot span the full event and are challenging to align in complex genomic contexts. Consequently, the same underlying variant may be inaccurately represented, split across multiple calls, or reported inconsistently by different methods (34). When it comes to the assembly, insufficient overlap between short fragments further complicates genome assembly and accurate breakpoint reconstruction, particularly in repetitive viral regions and highly amplified loci. As a result, segmental duplications and large repetitive elements are among the most frequently misassembled or unresolved regions in *de novo* assemblies based on short-read sequencing (35).

Therefore, these limitations highlight the need for sequencing technologies that can capture long-range genomic information, thereby providing a powerful framework for resolving integration structures and reconstructing complex structural variants.

### 2.2 Long-read whole genome sequencing

lrWGS, such as Pacific Biosciences (PacBio) and Oxford Nanopore Technologies (ONT), are two leading long-read sequencing platforms that present promising new directions and can be used to overcome the limitations of short-read sequencing. PacBio uses single-molecule real-time (SMRT) sequencing with circular consensus sequencing (CCS) to generate high accuracy (>99.9%), high fidelity (HiFi) reads that span from 10 to 25 kbp long (36) making PacBio well-suited for diploid genome reconstruction and SNP-level analyses. On the other hand, ONT infers the sequence based on the current flow through a nanopore as the DNA and RNA molecules pass through. ONT can produce ultra-long reads (from 10 kbp to >1 mbp) with moderate accuracy (95%-99% accuracy) (36–39). While both PacBio and ONT can generate reads that span multiple kilobases, they differ in error patterns, accuracy, assembly patterns, and quality. PacBio can achieve greater accuracy, lower error rates, and strong performance in haplotype-resolved assembly, while ONT can produce longer read lengths at a lower cost and is widely used for direct detection of epigenetic modifications (36).

In HPV research, PacBio long-read assembly has been successfully used to identify HPV integration breakpoints, construct complex models of HPV16 and HPV18 integration structures, and reveal structural variation (SV) in the SiHa and HeLa cervical cancer cell lines (40). Similarly, ONT long-reads identified clonal integration and various integration types present in a single tumor, which has given some insight into the complexity of HPV integration in cervical cancers (41). Long-read sequencing provides substantially longer flanking anchors on both sides of virus–host junctions, which improves alignment confidence and reduces ambiguity in read mapping, particularly in repetitive regions. As a result, breakpoint localization becomes more precise, minimizing false junction calls and misassignment of integration coordinates. In addition, the ability of long-reads to span large integration loci enhances confidence in copy number estimation by directly resolving tandem duplications and viral concatemers, thereby reducing uncertainties that commonly arise in short-read based analyses.

### 2.3 Target capture sequencing strategies

Target capture sequencing is an enrichment-based strategy used to selectively isolate genomic regions of interest before sequencing, thereby increasing coverage of low-abundance targets such as viral genomes and integration sites. Broadly, target capture approaches can be categorized into probe-based capture and real-time capture strategies. Probe-based capture methods rely on pre-designed synthetic probes that hybridize to target sequences, enabling selective enrichment before sequencing. Common implementations include commercial platforms such as Illumina capture panels, Twist Bioscience panels, nanopore Cas9-targeted sequencing (nCATS) (42), and Twist ONT (43), which are widely used in conjunction with short-read sequencing and can also be adapted for long-read platforms. These approaches provide high enrichment efficiency and are particularly effective for capturing viral genomes and virus–host junction regions. In contrast, adaptive capture methods dynamically enrich target sequences during sequencing without prior library enrichment. Nanopore-based approaches such as Readfish (44) and UNCALLED (45) perform real-time analysis of partially sequenced reads and selectively accept or reject DNA molecules based on alignment to a reference genome. These methods offer greater flexibility and do not require probe design.

Target capture strategies are particularly advantageous for studying viral integration, where viral sequences are often present at low abundance relative to the host genome. By increasing sequencing depth at integration sites, these approaches enable more accurate identification of virus–host junctions and facilitate downstream reconstruction of complex integration structures.

## 3 Viral integration detection as a specialized structural variant calling problem

Structural variants (SVs) are important driver alterations in several cancers. Structural variation can lead to gene amplification or deletion, changes to the cis-regulatory landscape, or the formation of splice variants and fusion genes. For example, genomic translocations result in the formation of fusion genes in several salivary gland cancers (46). These fusion genes are highly recurrent, salivary cancer type-specific, and necessary drivers of tumor growth (41, 47–49). To translate sequencing signals into explicit genomic rearrangements, dedicated structural variant detection tools are required. Advances in sequencing technologies have greatly improved the ability to characterize structural variation in cancer genomes. Structural variants, including deletions, insertions, inversions, duplications, and translocations, can disrupt genes, alter copy-number states, and reorganize chromosomal architecture. Viral integration can be regarded as a specialized form of structural variation because it creates a rearrangement at the integration site between two distinct reference spaces: the host genome and a viral genome. Consequently, many short-read and long-read based SV detection tools have been developed, benchmarked, and applied for cancer genome research (50–52).

There are two primary strategies for detecting SVs from whole genome sequencing data. The first uses an alignment-based approach whereby individual sequence reads are mapped to a reference genome to identify variants. The second approach uses hybrid strategies: first builds genome assembly contigs from individual reads and then aligns the contigs themselves against a reference to detect SV, including Dipcall (53), Smartie-sv (54), SVIM-asm (55), and PAV (56). In parallel, specialized tools have also been proposed for viral integration detection, including VirusFinder (57), ViralFusionSeq (58), VirusSeq (59), ViFi (60), SurVirus (32), and searcHPV (33) (**Table 2**), which are all mainly based on extracting pairs mapping partially to the host and partially to a virus, and cluster such pairs into viral integrations (32).

**Table 2:** Virus detection tools list.

Tools	Software	Recommended input
SearcHPV (33)	https://github.com/mills-lab/SearcHPV	Targeted capture DNA-seq
SurVirus (32)	https://github.com/kensung-lab/SurVirus	WGS/RNA-seq
ViralFusionSeq (58)	https://github.com/TF-Chan-Lab/ViralFusionSeq	DNA-seq/RNA-seq
VIcaller (110)	https://github.com/daweili-lab/VIcaller	WGS/RNA-seq
VirusSeq (59)	https://github.com/rosericazondekon/virusSeqPipeline	RNA-seq
VirusFinder (57)	https://bioinfo.uth.edu/VirusFinder/	WGS/RNA-seq/targeted sequencing
ViFi (60)	https://github.com/namphuon/ViFi	WGS/RNA-seq
FastViFi (111)	https://github.com/sara-javadzadeh/FastViFi	WGS/RNA-seq

### 3.1 Alignment-based SV detection tools

#### 3.1.1 Short-read alignment-based WGS SV detection tools

SrWGS-based SV detection mainly relies on alignment-related criteria: read-pair approaches assess the orientation and distance of paired ends; read-depth methods detect deletions or duplications based on divergences in mapping depth; split-read approaches leverage alignments that map over SV breakpoints DELLY, LUMPY, GRIDSS, Manta, and SvABA are state-of-the-art algorithms (61). However, using srWGS for SV detections has some limitations due to the length of read and the technology used for alignment, and some variants can’t be detected with short reads alone (61). Therefore, the use of long-read sequences is advisable, as they allow for a more comprehensive SV detection.

#### 3.1.2 Long-read alignment-based WGS SV detection tools

For lrWGS-based SV detection tools, alignment-based SV detection methods include PBHoney (62), Smartie-sv (63), Sniffles2 (64), DeBreak (65), and MAMnet (66). Some of them (Sniffles2, MAMnet, and DeBreak) have demonstrated relatively high and stable performance across different sequencing coverages (67). Assembly-based SV detection approaches(Dipcall, Smartie-sv, SVIM-asm, and PAV) are particularly effective at identifying large SVs, especially insertions, and are generally robust to variations in evaluation parameters and sequencing coverage, with few changes in the performance until the sequencing coverage dropped below 10-fold (67). Because assembly-based SV detection methods rely on assembled contigs as their primary input, the quality of structural variant identification is inherently dependent on the performance of the underlying genome assembly.

Several methods have been used to detect SVs in several cancer types (51, 68) with long-read WGS, identifying SVs that affect known oncogenes. One such study characterized the SV landscape in 21 colorectal cancer patients, including novel fusions affecting *RNF38* and *SMAD3 (*69). Another recent study by our group used nanopore sequencing to detect several novel structural variants in mucoepidermoid carcinoma (MEC) (70). These include a complex pattern of tumor-specific intermediate translocations, leading to the formation of *CRTC1*-*MAML2*, a common MEC driver gene fusion, as well as recurrent *TERT* promoter alterations associated with increased telomere length. By uncovering structural variants, including driver alterations, these methods are critical to understanding cancer genomics. We also used Sniffles2 on the synthetic long-read datasets described in the Methods to evaluate whether an alignment-based SV caller could recover HPV-human integration breakpoints. Sniffles2 detected many breakend (BND) events in which one side of the call mapped to a human chromosome and the other side mapped to the HPV reference (**Supplementary Tables 1–3**). These calls represent candidate viral-host junctions and illustrate the ability of long-read SV detection tools to identify integration sites when the viral genome is included in the reference.

### 3.2 Alignment-based and target-sequencing approaches SV detection tools for resolving viral integration structures

While long-read sequencing has substantially improved the detection of SVs in cancer, and modern SV detection tools have improved accuracy and reduced computation demands, there is still a need for tools with the ability to detect high confidence and accurate complex virus integration. HPV integrations into the genome often exhibit such complex structures; while such structures can sometimes result in a single, clean breakpoint, they more often involve multiple breakpoints, induce focal amplification, and generate fusion transcripts and complex structures that can span hundreds of kilobases (71). Compared to other genomic structural variations, such as deletions or transpositions, detecting viral integrations is more challenging and often computationally very expensive, producing inaccurate results. To address this issue, there are many tools designed especially for viral integration (**Table 2**). Existing short-read based methods for viral integration detection can be broadly divided into two main strategies. The first approach involves sequential alignment, where sequencing reads are initially mapped to the host (or viral) genome, followed by re-alignment of unmapped reads to the viral (or host) genome to identify chimeric sequences indicative of integration. ViralFusionSeq (58) is an example of a tool adopting this strategy. The second approach constructs a combined reference genome by concatenating host and viral sequences, to which sequencing reads are directly aligned. This strategy enables the detection of virus–host junctions through discordant and split-read signals, and is implemented in tools such as VirusSeq (59) and ViFi (60), as well as general-purpose structural variation detection methods.

A major challenge in viral integration detection lies in handling ambiguously mapped reads that align to multiple genomic locations. Existing approaches typically discard such reads, retain them only when supported by uniquely mapped reads, or rely directly on aligner-reported positions, thereby balancing sensitivity and specificity at the cost of missed structures or increased false positives. To address this limitation, SurVirus (32) introduces a clustering-based strategy inspired by transposable element detection. It iteratively groups reads supporting the same integration structure and identifies the most probable breakpoints in both host and viral genomes. This framework improves the handling of ambiguous reads and enhances detection accuracy, particularly in repetitive regions.

These strategies are typically designed for a broad range of virus types and rely on whole genome sequencing (WGS) with relatively uniform coverage, which can limit sensitivity for detecting rare viral integration structures. To address this limitation, targeted capture sequencing approaches have been developed, particularly for HPV, to enrich viral sequences and integration loci. While this strategy improves coverage at integration sites, it also necessitates highly sensitive and accurate computational methods for detecting viral–host fusion. To meet this need, searcHPV (33) was developed specifically for HPV-targeted capture sequencing data. SearcHPV analyzes targeted capture paired-end sequencing data by aligning reads to a concatenated human–HPV reference genome, enabling simultaneous detection of virus–host junctions. After duplicate removal and quality filtering, candidate fusion points are identified based on split reads and discordant read pairs. Reads supporting each candidate breakpoint are then extracted for local assembly, with overlapping read pairs merged before assembly to improve contiguity. The resulting contigs are aligned to the HPV genome to determine viral breakpoint locations. To resolve ambiguity, assembled contigs are classified based on their alignment patterns into directional groups representing different sides of the breakpoint. For each candidate fusion point, searcHPV selects one or two representative contigs to capture the most likely integration configuration, thereby refining breakpoint identification and improving structural interpretation.

## 4 Assembly tools and algorithms

Although long reads and targeted sequencing based approaches have substantially improved the sensitivity of integration site detection, they remain limited in their ability to resolve the complete structure of integration. Critical features such as large segmental rearrangements, tandem duplications, and complex virus–host–virus configurations are often not captured by breakpoint-centric methods. Given the limitations of mapping-based SV detection approaches, assembly-based methods are needed because longer assembled contigs can span complex viral–host junctions that are difficult to represent from read alignments alone. Hybrid SV detection tools described before that perform an assembly step before variant calling follow the same rationale. By reconstructing contiguous sequences across the integration locus, these approaches have the potential to resolve the orientation, ordering, and copy number of viral and host genomic segments and to recover the full integration structure. We will discuss assembly approaches from these three dimensions: (1) collapsed vs uncollapsed (phased), (2) reference-based vs reference-free (*de novo*), and (3) Overlap-Layout-Consensus (OLC) vs De-Bruijn-Graph (DBG).

### 4.1 Collapsed and uncollapsed (phased) frameworks for genome assembly

Long-read assembly methods can differ in how they represent genomic variation (**Table 3**). Long-read assembly strategies can be generally categorized into collapsed and uncollapsed (phased) frameworks. There are three types of parallel designs to choose from: “collapsed”, meaning reads from either haploid are fused to generate an overall contig; “semi-collapsed”, meaning alternative contigs are provided at locations where heterogeneity is presented, and “uncollapsed”, or phased assembly, meaning the contigs are presented so that the true homology information of the genome is preserved (72). For HPV integration reconstruction, collapsed and phased assembly strategies present distinct advantages and limitations.

**Table 3:** Assembly tools list.

Group	Tool	Software	Suggested input
Collapsed	Canu (76)	https://github.com/marbl/canu	PacBio CLR, ONT
	Shasta (77)	https://github.com/paoloshasta/shasta	ONT
	MBG (112)	https://github.com/maickrau/MBG	PacBio HiFi/CCS, ONT duplex reads
	Flye (73)	https://github.com/mikolmogorov/Flye	PacBio CLR/HiFi, ONT
	NextDenovo (78)	https://github.com/nextomics/nextdenovo	PacBio CLR, ONT
	Raven (75)	https://github.com/idaholab/raven	PacBio CLR, ONT
	Wtdbg2 (74)	https://github.com/ruanjue/wtdbg2	PacBio CLR, ONT
Semi collapsed	Falcon (79)	https://github.com/PacificBiosciences/FALCON	PacBio HiFi
	Peregrine (113)	https://github.com/cschin/peregrine	PacBio HiFi
	HiCanu (81)	https://github.com/marbl/canu	PacBio HiFi
Phased	verkko (114)	https://github.com/marbl/verkko	PacBio HiFi, ONT ultra-long
	hifiasm (80)	https://github.com/chhylp123/hifiasm	PacBio HiFi, ONT
	phasebook (115)	https://github.com/phasebook/phasebook	PacBio CLR/HiFi, ONT
	IPA	https://github.com/PacificBiosciences/pbipa	PacBio HiFi

Collapsed assembly (Flye (73), Wtdbg2 (74), Raven (75), Canu (76), Shasta (77), NextDenovo (78), et al.) integrates reads from both haplotypes into a single consensus representation, prioritizing structural continuity and overall genome completeness. Collapsed assembly integrates all supporting reads into a single consensus graph, which can enhance structural continuity and improve stability when integration alleles are unevenly represented or subclonal. By aggregating read support across haplotypes, collapsed approaches may more effectively preserve integration paths in cases of low allele frequency or heterogeneous structural configurations. However, graph simplification procedures may collapse tandem viral copies or repetitive host–virus segments, potentially obscuring precise copy number and allelic structure.

Semi-collapsed assembly (Falcon (79) et al.) is designed for representing heterozygous regions in complex genomes. It can output heterozygous regions in diploids. The main difference between semi-collapsed and collapsed is the treatment of assembly graph bubbles. Semi-collapsed assemblies will keep all possibilities, select the primary contigs with high continuity, and put the other side as alternative contigs, which are fragmented and incomplete.

In contrast, phased assembly [Hifiasm (80), HiCanu (81), et al.] attempts to separate reads into haplotype-specific paths, preserving allele-specific variation and enabling haplotype-resolved reconstruction. Many of these methods were originally developed for high-accuracy long-read data such as PacBio HiFi sequencing and rely on high-confidence heterozygous SNP signals together with approximately balanced diploid coverage to guide haplotype separation. When HPV integration occurs in a balanced, allele-specific (1:1) manner, phased assembly may more accurately separate host-only and host–virus alleles and preserve haplotype-specific structural information. Nevertheless, HPV integrations frequently exhibit focal amplification, or subclonal representation. Under such conditions, the assumptions of balanced diploidy and SNP-driven partitioning may not fully apply. Haplotype partitioning may therefore fragment integration-supporting reads or reduce effective coverage at complex junctions, particularly when structural variation rather than SNP heterozygosity dominates the local signal. Consequently, the performance of phased assembly may depend strongly on integration clonality, coverage balance, and local structural complexity.

### 4.2 Reference-based and reference-free (*de novo*) assembly

Assembly approaches can be broadly categorized into reference-based and *de novo* strategies. Reference-based, also called alignment-based approaches, leverage read mappings to a reference genome to guide contig reconstruction, like RGAAT (82). However, because they rely on initial read alignments, these approaches are inherently constrained by mapping accuracy and may not be suitable to fully resolve complex, repetitive, or highly rearranged integration structures. Furthermore, there is a distinct ‘reference bias’ whereby alleles and genome structure present in the reference genome tend to have a disproportional representation, though advances in pangenome construction have somewhat alleviated this (83–85). In contrast, reference-free (*de novo*) assembly reconstructs sequences directly from reads without reliance on a reference genome, enabling more complete recovery of novel sequences and complex structural configurations. As a result, *de novo* assembly has emerged as a powerful strategy for resolving intricate viral–host integration architectures, particularly in the context of long-read sequencing (86, 87).

High-quality *de novo* genome assembly from long-read data remains computationally intensive, whereas faster assemblers often sacrifice contiguity or accuracy. This trade-off between computational efficiency and assembly quality continues to pose a major challenge in large-scale genomic studies (67). Because this paper focuses on virus integration reconstruction, we primarily focus on reference-free (*de novo*) assembly methods from the perspective of discovering novel sequences.

### 4.3 Algorithms for *de novo* assembly

The effectiveness of *de novo* assembly largely depends on its underlying algorithmic framework, which determines how reads are organized and assembled into contiguous sequences.

#### 4.3.1 Overlap-layout-consensus and string graph

In brief, the OLC approaches consist of three major steps: building an overlap graph, constructing contigs, and finding consensus of nucleotides to finalize the sequence of each contig (88). In the first step, the goal is to find overlapping patterns between any pair of reads and create an overlap graph where each node stands for a read and each directional edge stands for reads-overlapping connection. Different OLC algorithms utilize a variety of alternative matching methods to account for sequencing errors, mismatches, and gaps. The all-to-all pairwise alignment is the most time-consuming and memory-intensive step, as both increase quadratically with the number of reads, bottlenecking the applicability of OLC-based tools on larger genomes. Next, the OLC method prunes the overlap graph and consolidates the edges by removing the abruptly ended paths and the inferable edges, resulting in a layout of continuous paths of nodes (which constructs the contigs) and sections of bridging parts of the graph. The bridging parts represent the sequencing-error-enriched, repetitive parts of the genome, which are usually left out by the OLC tools. The last step is finding consensus of the contigs by piling up the contig-supporting reads and getting the majority vote of each position.

Although the classical OLC paradigm constructs a full overlap graph (OLG), modern long-read assemblers rarely adopt the raw OLG representation due to its quadratic complexity and redundant transitive overlaps. Instead, string graph formulations eliminate transitive edges and significantly reduce graph density while preserving assembly information, thereby improving scalability and repeat resolution (89, 90). Several widely used long-read assemblers are based on the OLC paradigm or its string-graph variants, including Canu, NextDenovo, Raven, and Shasta.

#### 4.3.2 De Bruijn graph

De Brujin graph algorithms segment reads into shorter k-mers and build graphs with k-mers as nodes and directional edges pointing to neighboring k-mers in reads (91). The DBG then tries to infer the whole genome by constructing an Eulerian walk through the graph. Compared to OLC, no consensus calling step nor pairwise alignment is needed in DBG, making it computationally efficient in two parts: the computational time increases linearly with the total length of reads and is up bounded by the size of the genome regardless of average sequencing depth. To accommodate long-read data with elevated error rates, modified DBG formulations have also been developed. These include repeat graphs, minimizer-based graphs, and fuzzy de Bruijn graph representations (74, 92). For example, Wtdbg2 constructs a “fuzzy” de Bruijn graph by grouping sequences into bins and connecting them using minimizer-based anchors rather than exact k-mer matches. This design increases tolerance to sequencing errors while preserving computational efficiency.

#### 4.3.3 Applying OLC and DBG to whole genome assembly

When applying these algorithms to assemble genomes using real sequencing data, we see major challenges arise: the human genome is diploid, and natural heterozygosity needs to be considered; >40% of the human genome is repetitive, composed of repeated elements, short tandem repeats, and regional duplications; all sequencing technologies produce errors: the accuracy ranges 95%-99.99% depending on the technology.

OLC can tolerate small levels of heterozygosity by toning the matching algorithm to allow mismatches and small gaps, whereas DBG is typically less forgiving of errors because they result in many k-mer nodes that only appear once in the dataset and then are typically pruned. Low-quality read filtering and pre-assembly error correction procedures are usually necessary for DBG. Both algorithms tend to exclude unresolvable repeats and output only clean long contigs. The naturally repetitive structure resulting from HPV integrations, especially structures with complex localized genomic rearrangements, thus poses great challenges for these algorithms. HPV integrations can generate repeats that are longer than the average read length, depending on the sequencing method, as the HPV genome alone is about 8,000 base pairs long. It is not uncommon to see evidence of a long repetitive sequence consisting of an integrated HPV sequence and flanking human genome sequence that is duplicated multiple times at local regions (41). As a result, precise virus–host breakpoints are not always fully resolved, and multi-copy integration structures are often partially collapsed or fragmented in assembled contigs. In addition, many assembly algorithms are designed to simplify redundant graph paths, which may merge duplicated viral or host segments and obscure true copy-number states. These challenges highlight the limitations of current long-read assemblers in accurately reconstructing complex viral–host integration architectures.

## 5 Evaluating reconstruction of HPV integration structures using genome assembly: a case study

### 5.1 Study design and overview

To evaluate the limitations of existing assembly methods in reconstructing HPV integration structures, we designed a benchmarking framework (**Figure 2**). Due to the uncertainty of the true integration structures in real sequencing data and for the convenience of accurate calculation, we created three synthetic datasets representing HPV integration with increasing levels of structural complexity to evaluate whether current assembly tools can reconstruct complete integration structures. In addition, to preserve and recapitulate the true HPV integration architecture, the integration structures used in this study were derived from previously published models (41) as well as our prior research on HPV integration sites (33).

**Figure 2:**
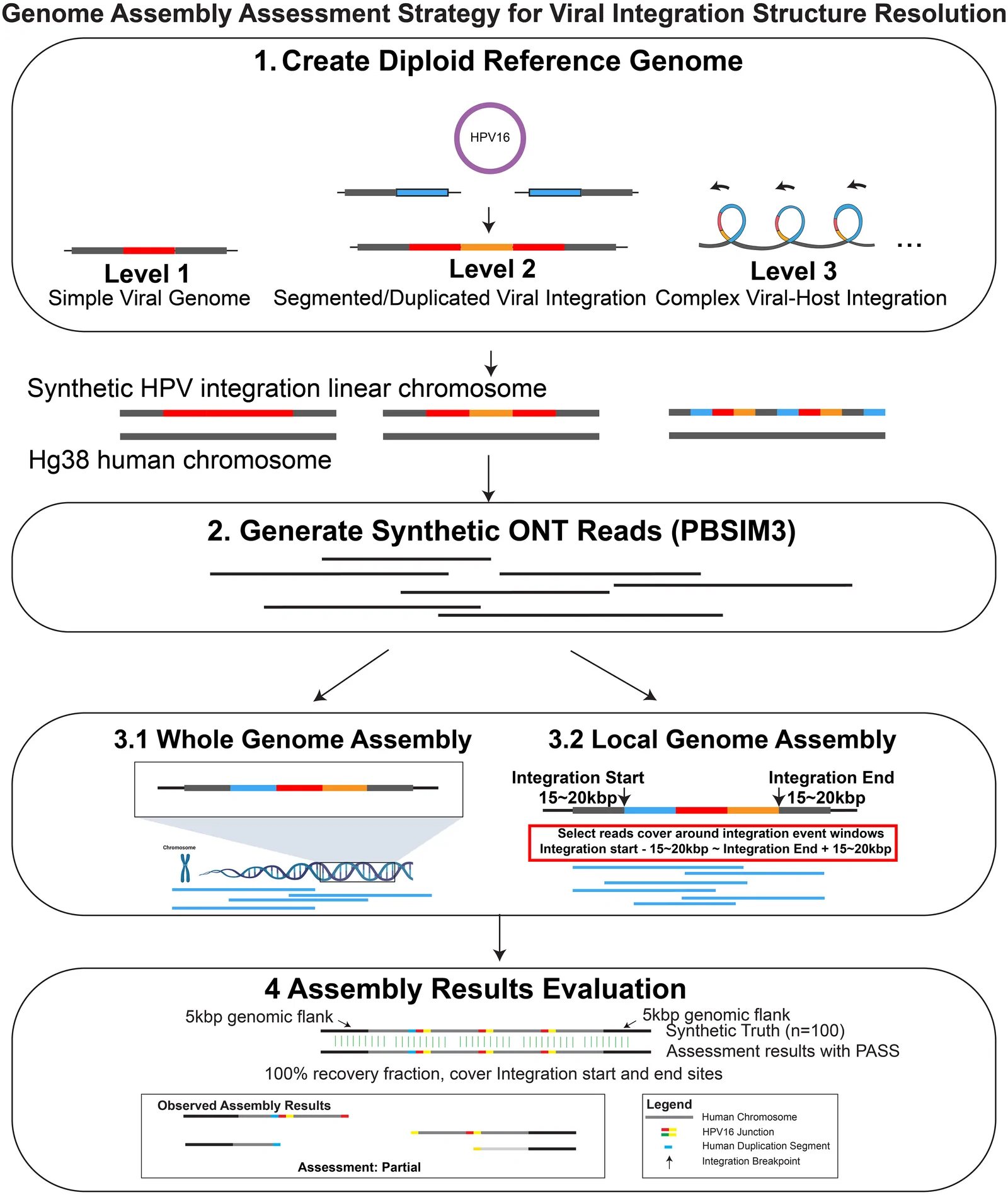
Framework for synthetic HPV integration read simulation, assembly, and reconstruction assessment. Synthetic HPV integration references were generated to represent three levels of increasing integration complexity, ranging from simple viral insertions to duplicated viral segments and highly rearranged viral-host structures. For each level, 100 integration sites were simulated by inserting representative HPV integration structures into selected hg38.p14 genomic loci, and the host–HPV reference genome was used as haplotype 1, while the unmodified hg38.p14 genome was used as haplotype 2. PBSIM3 was then used to simulate ONT long-read datasets from haplotype 1, haplotype 2, and additional windows surrounding each integration site. The simulated reads were assembled using both whole genome and local assembly strategies with six long-read assemblers: Raven, Flye, Wtdbg2, Shasta, NextDenovo, and Canu. For local assembly, reads overlapping breakpoint-centered windows and HPV were extracted before assembly. Assembled contigs were aligned to structure-specific synthetic truth references using Minimap2, and reconstruction performance was evaluated based on recovered junctions, truth-reference coverage, aligned identity, and PASS classification (see Methods). Some schematic elements were created with BioRender.com.

In this benchmarking framework, we primarily focused on ONT long-read data and collapsed assembly strategies. We further emphasized collapsed assembly frameworks based on both biological and methodological considerations. Most semi-collapsed and uncollapsed (phased) assembly frameworks were originally developed for high-accuracy long-read platforms, particularly PacBio HiFi sequencing, where accurate single-nucleotide variant (SNV) detection enables reliable SNP-driven haplotype partitioning. In contrast, our primary objective was to reconstruct the overall integration structures rather than to resolve fine-scale haplotype variation.

### 5.2 Methods

#### 5.2.1 Create synthetic HPV integration references

As described above, integrated HPV genomes can be categorized as simple or complex integration patterns. Importantly, many previous studies have shown that HPV integration is often more complicated than a single linear viral insertion (8, 41, 93). Complex integration events may include duplication or inversion of viral fragments, rearrangement of viral segments, and, in some cases, co-amplification of adjacent human genomic sequences. For evaluating assembly performance across integration structures with varying structural complexity, we selected representative samples from these three categories and treated them as Level 1, Level 2, and Level 3, respectively (**Figures 3B–D**), representing increasing levels of integration complexity. Level 1 consists of simple HPV integrations containing a single viral segment (**Figure 3B**: Structures A and B). Level 2 incorporates duplicated HPV segments within the host genome (**Figure 3C**: Structures C and D), with Structure C representing a transitional configuration between Levels 1 and 2. Level 3 is characterized by multiple novel HPV–human, HPV–HPV, and human–human junctions (**Figure 3D**: Structures E, F, and UDSCC2), where Structure F represents an intermediate form between Levels 2 and 3. Together, these structures illustrate a progressive increase in integration complexity from simple insertions to highly rearranged virus–host architectures. Furthermore, to better reflect the diversity observed in real-world HPV integration structures, we used a fragment-selection window to include additional simulated structures based on validated integration structures. Specifically, the length of each inserted HPV and host fragment was preserved as was the chromosome of insertion. Also, start positions within the HPV genome were randomly selected, with a bias toward the E6 and E7 regions, and host fragments were selected near the integration sites.

**Figure 3:**
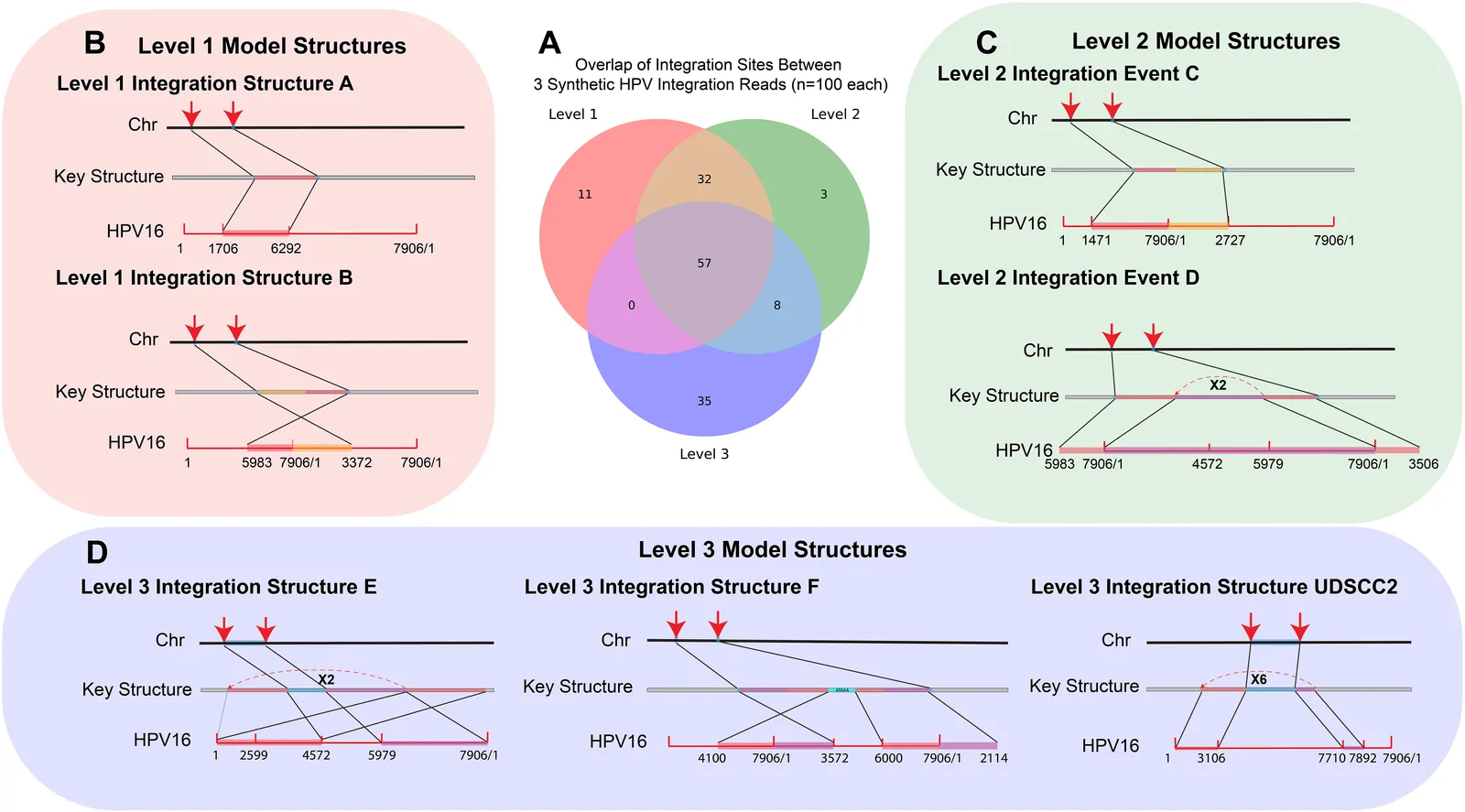
Synthetic HPV integration structures in Level 1 to Level 3. **(A)** Comparison of integration sites across the three synthetic read datasets, highlighting overlapping and unique HPV integration loci among the datasets. **(B–D)** Synthetic HPV integration structure models used to generate synthetic reads with increasing levels of HPV integration complexity. The three models represent progressively more complex viral-host architectures, ranging from simple HPV-host junctions to rearranged and repeat-containing integration structures.

These integration structures were then inserted into HPV integration sites previously identified in Target Capture Sequences datasets using the in searcHPV paper (33), together with ~10–50 randomly selected genomic sites (**Figure 3A**). Problematic regions were excluded from the hg38 genome using publicly available annotation files, including assembly gaps from the UCSC Genome Browser (94), ENCODE blacklist regions (95), and telomere-proximal regions (<500 kbp from chromosome ends). Because integration structures may also cause deletions near integration sites, to ensure the authenticity of the insertion point, each insertion site was spaced by at least the size of the deletion, yielding a total of 100 integration sites. We chose one of the most common and high-risk HPV types: HPV16, and finally generated three linear human chromosome reference sequences (version hg38.p14) containing HPV16 (96) integrations to represent haplotype 1 (the host–HPV reference genome), while the unmodified hg38.p14 reference genome was used to represent haplotype 2. To accelerate the assembly process, only chromosomes with viral integrations were included in the HPV16 integration reference sequence. The integration sites and structure lists are detailed in **Supplementary Tables 4**–**6**.

#### 5.2.2 Simulation of long-read sequencing data

After generating linear HPV integration reference sequences, we used PBSIM3 (97) to simulate synthetic ONT long reads. Due to the complexity and uneven coverage near integration sites and to ensure sufficient read coverage for each integration structure, we generated extra windows of ±30,000 bp around each integration site. For level 3, a wider window of ±80,000 bp was applied due to the higher copy number and increased structural complexity. In total, reads were generated from three components: (1) haplotype 1 (the host–HPV reference genome), (2) haplotype 2 (hg38.p14), and (3) integration site windows, with a read-depth ratio of 3:3:1.

Previous studies for long-read assembly and structural variant detection typically evaluate performance across a broad range of sequencing depths (e.g., 5×–50×) (67), reflecting the trade-off between sequencing cost and reconstruction accuracy. Considering both cost–accuracy trade-offs and the minimum coverage requirements recommended by long-read assemblers such as Flye and Wtdbg2, we therefore selected the mean sequencing coverage of 30× (15× haplotype 1, 15× haplotype 2) as the primary background depth and increased the coverage to 35× around integration sites (additional 5x integration site windows), representing more realistic and widely adopted coverage levels for evaluating assembly accuracy and HPV integration recovery (41, 98–100). Read-length parameters were selected based on both ONT-reported sequencing characteristics and previously published long-read HPV integration study (41), which typically generate reads in the 10–25 kbp range. To simulate realistic sequencing conditions, we set the mean read length (LENM) to 10 kbp and further calibrated the standard deviation (LENS = 6 kbp), minimum read length (LENMIN = 3 kbp), and maximum read length (LENMAX = 1 mbp) according to the empirical read-length distribution of our ONT sequencing data.

#### 5.2.3 Extract reads for local assembly

For local assembly, reads were selected if any portion of their alignment using Minimap2 (101) overlapped a 15 kbp window (using 20 kbp for NextDenovo and Raven) centered on the host breakpoint. The extraction window size was selected empirically for each assembler based on the number of successfully reconstructed (PASS) integration events. We observed that most assemblers performed better with a 15 kbp window, whereas NextDenovo and Raven achieved more PASS events with a 20 kbp window (**Supplementary Tables 12**, **13**).

#### 5.2.4 Assembly tools and parameter settings

We primarily focus on Raven (75) (v1.8.3), Flye (73) (v2.9.6), Wtdbg2 (74) (v2.5), Shasta (77) (v0.14.0), NextDenovo (63) (v2.5.2), and Canu (76) (v2.3), which have been widely used in genome assembly and have been updated recently. We evaluated both whole genome assembly and local assembly. All assemblies’ results were aligned to haplotype 1 (the host–HPV reference genome) using Minimap2 (101).

#### 5.2.5 Evaluation of assembly results

We first used Minimap2 (101) to map the reads to the HPV16 reference. Any read with at least one alignment segment mapped to HPV16 was flagged and extracted. Within these selected reads, for each simulated integration structure, the best contig alignment was selected by prioritizing the number of recovered junctions, truth-reference coverage, aligned sequence identity, mapping quality, and alignment block length. Selected assembly contigs were aligned to the host–HPV reference genome using Minimap2 in PAF format. Each synthetic HPV truth reference consisted of the simulated integration sequence with flanking host sequence on both sides (5 kbp). Aligned sequence identity was calculated by number of matching bases/alignment block length. Recovery fraction was defined as the fraction of the structure-specific synthetic HPV truth references covered by the best alignment: (target_end - target_start)/target_length. Because the structure-specific synthetic HPV truth references included the host 5 kbp flanking sequence, the recovery fraction represents coverage of the full local reconstructed structure reference, including both flanks and the inserted sequence. Left and right junction recovery were determined by whether the best alignment spanned the expected junction coordinates within a 50 bp window. Events were classified as PASS if both integration start and integration end junctions were recovered, the aligned identity was at least 95%, and the recovery fraction was at least 80% (**Figure 4A**). Events with partial alignment evidence that did not meet PASS criteria were classified as PARTIAL, and structures with no contig alignment were classified as MISS. A structure was considered to have a corresponding aligned contig if at least one PAF record mapped an assembled contig to its structure-specific truth reference. The resolution rate was calculated as the percentage of structures with at least one such contig alignment, corresponding to structures classified as PASS or PARTIAL rather than MISS.

**Figure 4:**
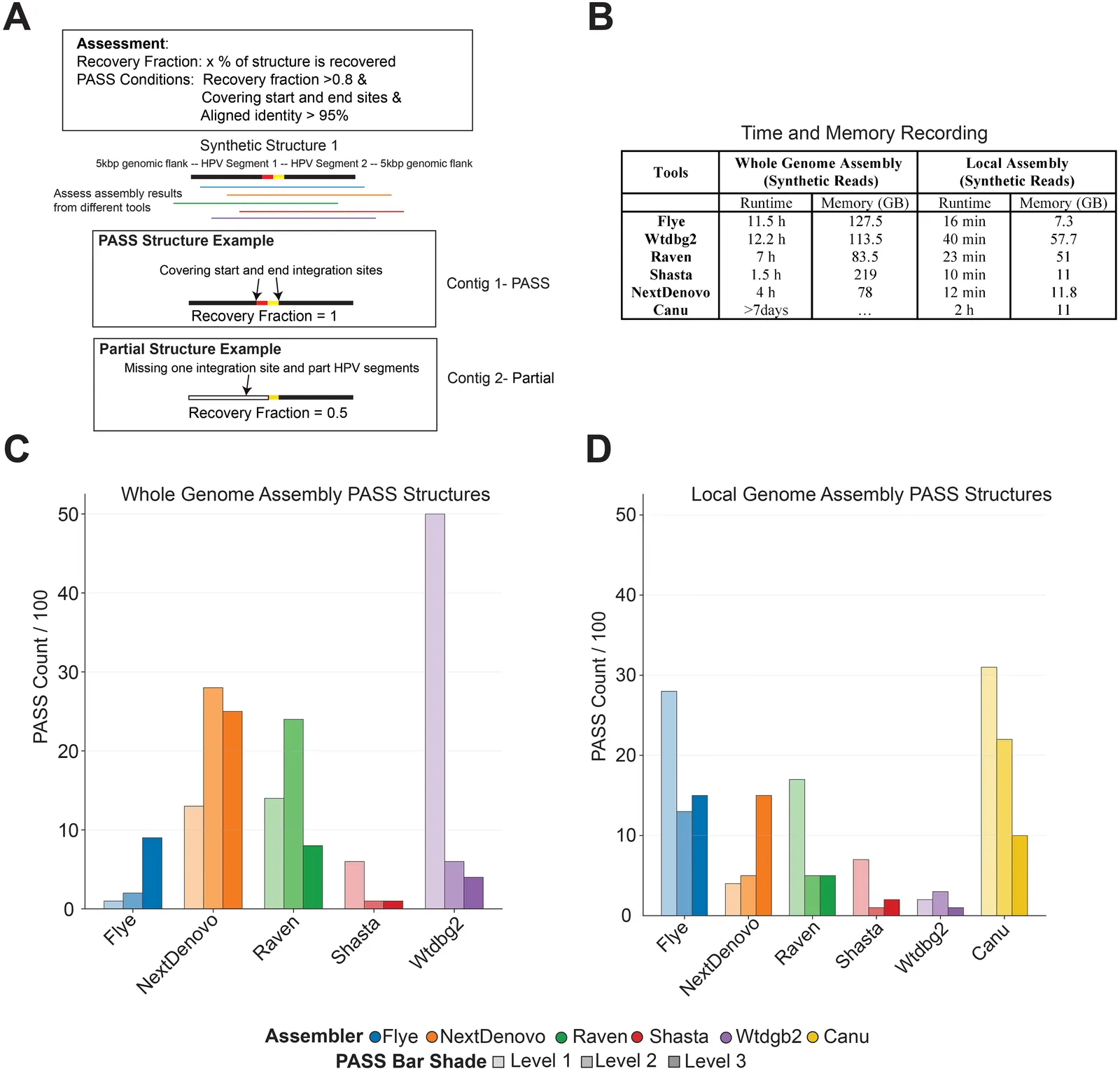
Assembly results and comparison between whole genome assembly and local genome assembly. **(A)** Assessment methods for assembly results. Contigs are considered PASS if both integration start and integration end junctions were recovered, the aligned identity was at least 95%, and the recovery fraction was at least 80%. **(B)** Computational resource usage, including runtime and memory consumption, for each assembly tool under whole genome and local assembly settings. **(C)** PASS rate of whole genome assembly results under predefined accuracy criteria. A result was considered passing if both the integration-start and integration-end junctions were recovered, the aligned identity was at least 95%, and truth-sequence coverage was at least 80%. (Canu is excluded due to high computational time). **(D)** PASS rate of local assembly results under the same accuracy criteria used in panel **(B)** Source data for this image are provided in **Supplementary Tables 10, 11**.

To characterize the assembly output statistics, including contig count and length distribution, were calculated using seqkit stats (102). Assembled contigs were aligned to the host–HPV reference genome using Minimap2, and alignment files were processed with samtools (103) to calculate N50 and physical coverage of contigs. Contigs containing HPV sequences were identified based on alignments to the HPV reference (**Supplementary Tables 7**, **8**). To evaluate potential redundancy and overlap among contigs, an all-versus-all contig alignment was performed using Minimap2. For each non-self-alignment, alignment length, sequence identity, and coverage proportions relative to both query and target contigs were calculated. Query coverage (qcov) and target coverage (tcov) were defined as the aligned span divided by the total length of the query and target contigs, respectively. To avoid counting redundant reciprocal alignments, only the best alignment per contig pair (defined as the alignment with the longest aligned length and highest identity) was retained. Based on alignment length, sequence identity, and contig coverage, overlapping contig pairs were classified into three categories: (i) duplicate contigs (alignment ≥ 2 kbp, identity ≥ 85%, and ≥ 80% coverage of both contigs), (ii) containment pairs (alignment ≥ 4 kbp, identity ≥ 85%, and ≥ 80% coverage of one contig but not the other), and (iii) shared-repeat pairs (alignment ≥ 8 kbp, identity ≥ 75%, and < 50% coverage of both contigs). The number of contig pairs in each category was summarized to assess assembly redundancy and repeat-associated overlap. In our assemblies, only duplicate and containment were observed.

### 5.3 Results

#### 5.3.1 Synthetic reads

The synthetic dataset was constructed with three levels of increasing integration complexity with 100 integration structures in each (see Methods). Level 1 consisted of 50 structures A and 50 structures B, incorporating 52 randomly selected genomic insertion sites and 48 previously reported HPV integration sites. Level 2 included 50 structures C and 50 structures D, with 44 random insertion sites and 56 previously reported HPV integration sites. Level 3 comprised 34 structures E, 33 structures F, and 33 UDSCC2 structures, incorporating 80 random insertion sites and 20 previously reported HPV integration sites. Most genomic insertion sites were shared across the three complexity levels (**Figure 3D**). This design minimizes potential site-specific biases and allows reconstruction performance to be evaluated primarily as a function of integration structural complexity rather than differences in genomic insertion context.

To ensure that the input reads sufficiently cover HPV integration regions, we evaluated multiple read-level features, including the number and the N50 of HPV-Human chimeric reads, effective HPV coverage, and the mean sequencing depth across all reads. These features are summarized in **Table 4**. Across the three synthetic samples (Level 1–Level 3), both the number of HPV split reads and effective HPV coverage increased progressively as HPV copy number increased. However, the N50 of HPV chimeric reads remained stable at ~14–15 kbp, and the whole-read mean depth was comparable across samples. Here, contig statistics were calculated using only contigs containing HPV sequences, as identified by alignment to the HPV reference.

**Table 4:** Synthetic reads features.

Reads ID	HPV split reads counts	HPV reads N50	HPV effective coverage
Level 1	2027	15049	910.152
Level 2	3310	14425	3608.447
Level 3	6503	14054	3156.719

HPV effective coverage = sum (aligned reference bases on HPV)/HPV_LEN.

#### 5.3.2 Assembly results for synthetic reads

##### 5.3.2.1 Whole genome assembly

Using whole genome assembly, we first evaluated the ability of HPV integrations to be reconstructed by 6 widely used collapsed assembly tools (Flye, Wtdbg2, Raven, Shasta, NextDenovo, and Canu) across increasing structural complexity. Canu was excluded from whole genome assembly analysis due to its substantially higher computational time, which exceeded practical limits of this benchmarking framework.

Overall, assembly performance varied considerably across assemblers and integration complexity levels (**Supplementary Table 10**) with resolution rate and average recovery fraction for 100 structures per level ranging from 20% to 70%. Reconstruction average recovery fraction generally decreased as structural complexity increased, indicating that complex HPV integration patterns remain challenging to resolve using whole genome assembly approaches. For simple integration structures (Level 1), Wtdbg2 achieved the highest resolution rate (80%) and recovery fraction (0.7), whereas the remaining assemblers typically achieved average recovery fractions between 0.4 and 0.6. For moderately complex structures (Level 2), NextDenovo and Raven showed the best performance, reaching average recovery fractions of approximately 0.6 and resolution rates approaching 80%. For the most complex structures (Level 3), resolution rates remained relatively high for Flye, NextDenovo, and Raven (~80%); however, many reconstructed contigs contained only partial HPV sequences without fully resolving the host–virus structure. Consequently, average recovery fractions declined substantially when comparing Level 1 and Level 2. When applying the PASS rate (See Methods), the highest PASS rate was observed for Wtdbg2 in Level 1 structures, reaching approximately 50%, while PASS rates for more complex structures remained substantially lower (less than 30%) (**Figure 4C**). These results suggest that although some assemblers can detect HPV-containing contigs, accurate reconstruction of complete integration structures remains challenging, particularly for complex structures.

##### 5.3.2.2 Local genome assembly results

We next evaluated a local assembly strategy in which reads mapped to HPV and covered a 15–20 kbp region centered on the host breakpoint (as identified with Minimap2; see Methods) were provided as input to each assembler. Overall, most assemblers showed higher average recovery fractions under the local assembly framework (**Supplementary Table 11**). In Level 1, Flye and Canu demonstrated the strongest overall average performance (0.8 average recovery fraction), followed by NextDenovo and Raven (0.7 average recovery fraction). In Level 2, Canu got the highest resolution rates 78% and 0.7 average recovery fraction. In Level 3, NextDenovo got the highest average recovery fraction (0.5). We then tested the PASS rate among all assemblers. Canu achieved the largest number of PASS structures for both Level 1 (31) and Level 2 (22) integrations, whereas Flye and NextDenovo recovered 15 PASS structures in Level 3 (**Figure 4D**). When comparing local assembly results to whole genome assembly results, most of them have an increasing average recovery fraction of 0.1-0.5 (**Supplementary Table 11**). In addition, local assembly required substantially less runtime and memory than whole genome assembly (**Figure 4B**), making it a more computationally efficient approach. Despite these improvements, the overall PASS rate remained below 35% across all complexity levels, indicating that even targeted local assembly struggles to fully reconstruct a substantial proportion of HPV integration structures. Together, these results suggest that local assembly improves both computational efficiency and reconstruction accuracy, but further methodological advances are still needed in local assembly tools to accurately resolve highly complex integration structures.

#### 5.3.3 Assembly results for cell line samples

UMSCC47 is a head and neck squamous carcinoma cell line of Type 2 integrated HPV16 (104–106). Previous studies which explored this cell line using Oxford Nanopore sequencing, 10X Genomics linked-read sequencing, target capture sequencing, WGS, and real-time PCR (33, 107) successfully discovered that this cell line contains HPV integration sites, and suggested the presence of HPV integration structures with high copy number. However, based on inspection of the alignment BAM files and integration sites identified by searcHPV, additional complex integration structures appear to be present that have not yet been fully resolved. To further investigate these structures, we applied these six assembly tools to reconstruct the HPV integration region on chr3 (~189.88–189.90 mbp) using Nanopore sequencing datasets. As with the synthetic read benchmarking, we applied both whole genome and local genome assembly approaches to UMSCC47.

Across assemblers, contigs spanning the HPV16 integration locus were consistently recovered with concordant host–virus breakpoints, indicating reliable detection of the integration site. However, accurate reconstruction of the internal viral structure remained challenging, as several assemblies showed shortened or fragmented viral segments consistent with partial collapse of tandem HPV repeats. Targeted local assembly improved the resolution of these complex repeat structures. For example, Canu, NextDenovo, and Wtdbg2 successfully reconstructed the 2–4 tandem HPV repeat architecture together with the chr3 segment at the integration locus, demonstrating the potential advantage of local assembly strategies for resolving structurally complex viral insertions (**Figure 5**). Notably, real-time PCR and WGS-based copy number estimates indicate substantially higher HPV copy numbers in UMSCC47 (21.1 by real-time PCR; 47 by WGS) (107). Notably, because UMSCC47 is a tumor-derived cell line, HPV copy number may vary across passages and subclonal populations, potentially contributing to differences among reported estimates. Technical factors, including DNA preparation, sequencing depth, and copy number estimation methods, may further influence the apparent HPV copy number. Together, these biological and technical considerations suggest that current assemblies likely underestimate the true structural complexity of the viral integration locus. Accurate reconstruction of large tandem viral arrays remains constrained by both sequencing limitations and assembly algorithm performance, underscoring the need for continued improvements in both sequencing technologies and assembly methodologies.

**Figure 5:**
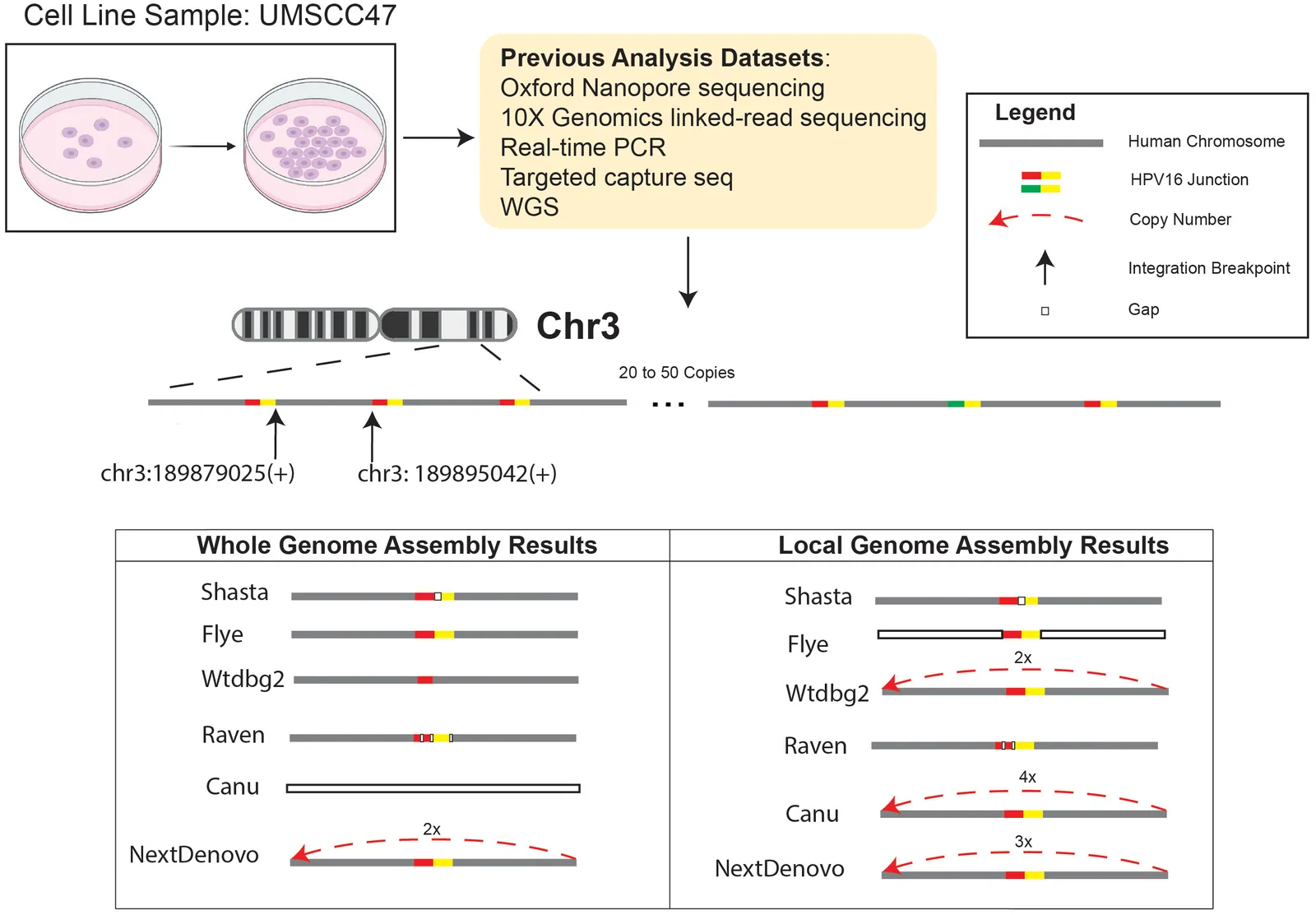
Comparison of whole genome and local genome assembly results for HPV16 integration reconstruction in the UMSCC47 cell line. UMSCC47 is an HPV-positive head and neck squamous cell carcinoma cell line with a previously characterized HPV16 integration locus on chromosome 3. Prior evidence from Oxford Nanopore sequencing, 10X Genomics linked-read sequencing, real-time PCR, targeted capture sequencing, and WGS supports a high-copy HPV16 integration structure at chr3:189879025–189895042, with an estimated 20–50 copies. The upper panel summarizes the experimentally supported HPV16 integration region and breakpoint positions. The lower panel compares reconstruction results from six long-read assemblers using whole genome assembly and local assembly strategies. Human genomic segments are shown in gray, HPV16 junctions are shown in red and yellow, copy-number structures are indicated by red dashed arcs, integration breakpoints are marked by black arrows, and gaps are indicated by open boxes. Local assembly improved recovery of high-copy HPV16-associated structures for several assemblers, particularly Wtdbg2, Canu, and NextDenovo, compared with whole genome assembly. Some schematic elements were created with BioRender.com.

## 6 Conclusion

Virus integration is a driver of genome instability and oncogenesis in multiple human cancers. Although long-read sequencing technologies now enable direct observation of viral–host junctions and long-range rearrangements, accurately reconstructing complete viral integration structures remains challenging. Through systematic evaluation using both synthetic and real long-read datasets, we show that current SV callers can detect viral–host breakpoints but fail to represent full integration architectures, while assembly-based approaches remain limited by breakpoint inaccuracy, fragmentation, copy-number collapse, and sequencing depth. These results reveal a fundamental gap between the complexity of viral integration structures and the assumptions embedded in current assembly and SV detection tools. Future original research studies including additional viral-positive cell lines and tumor datasets will be needed to systematically evaluate assembler performance across diverse viral integration patterns and biological contexts. Our findings highlight the urgent need for integration-aware assembly strategies and a better method that preserves copy number and alternative paths, along with visualization frameworks tailored to viral–host rearrangements. Such advances will be essential for achieving accurate and biologically faithful reconstruction of HPV integration structures and for advancing our understanding of viral oncogenesis in cancer genomes.

## Supplementary material

Supplementary Tables 1–3 Sniffles2 results for three synthetic samples.

Supplementary Tables 4–6 Level 1 to Level 3 HPV integration structures list.

Supplementary Table 7 Whole Genome Assembly Contig Features.

Supplementary Table 8 Local Genome Assembly Contig Features.

Supplementary Table 9 Runtime and Memory Usage.

Supplementary Tables 10, 11 Source data for assembly results assessment and **Figure 4C**.

Supplementary Tables 12, 13 Local assembly assessment results for 20 kbp windows and 15 kbp windows.
